# Improving Anesthesia Start Time Documentation Through a Departmental Education Initiative at Yale New Haven Hospital, New Haven, United States

**DOI:** 10.7759/cureus.54351

**Published:** 2024-02-17

**Authors:** Michael H Warren, Sumarth Mehta, Lena Glowka, Octavio Goncalves, Elena Gutman, Robert B Schonberger

**Affiliations:** 1 Department of Anesthesia, Critical Care and Pain Medicine, Massachusetts General Hospital, Boston, USA; 2 Department of Anesthesiology and Pain Management, University of Texas Southwestern Medical Center, Dallas, USA; 3 Department of Anesthesiology, Yale School of Medicine, New Haven, USA

**Keywords:** anesthesia start time, process improvement, reimbursement, healthcare economics, anesthesia information management systems, billing compliance, anesthesia documentation, quality improvement, anesthesiology

## Abstract

Background

Reimbursement for anesthetic services in the United States utilizes a formula that incorporates procedural and patient factors with total anesthesia time. According to the Centers for Medicare & Medicaid Services and the American Society of Anesthesiologists, the period of billable time starts when the anesthesia practitioner assumes care of the patient and may include transport to the operating room from the preoperative holding area. In this report on a quality improvement effort, we implemented a departmental education initiative aimed at improving the accuracy of anesthesia start-time documentation.

Methods

Utilizing de-identified, internal data on surgical procedures at Yale New Haven Hospital (YNHH), New Haven, United States, the difference between documented anesthesia start and patient in-room time was determined for all cases. Those with a difference between 0-1 minute were assumed “likely underbilled,” and the total revenue lost for these cases was estimated using a weighted average of institutional reimbursement per unit of time. A monthly, department-wide educational email was then introduced to inform practitioners about the guidelines around start-time documentation, and the percentage of “likely underbilled” cases and lost revenue estimates trended over a one-year period.

Results

Baseline data in December 2020 showed that of the 6,877 total surgical cases requiring anesthesia at YNHH, 55.1% (N=3,790) had an anesthesia start to in-room time of 0-1 minute, which were considered “likely underbilled.” The average start-to-in-room time for properly recorded cases (44.9%, N=3,087) was 4.42 minutes. The baseline revenue lost in December 2020 for underbilled cases was estimated at $52,302. Over the one-year quality improvement initiative, the proportion of underbilled cases showed a downward trend, decreasing to 29.2% of total cases by November 2021. The estimate of revenue lost due to underbilling also showed a downward trend, decreasing to $29,300 in November 2021.

Conclusion

This quality improvement study demonstrated that a relatively simple, department-wide educational email sent monthly correlated with an improvement in anesthesia start-time documentation accuracy and a reduction in estimated revenue lost to underbilling over a one-year period.

## Introduction

In the United States, reimbursement for anesthetic services utilizes a formula that includes base units and time units. Base units are standardized and depend on the type and complexity of the surgery along with patient characteristics, while time units are determined by the total length of time that anesthesia was provided. A single time unit equates to 15 minutes of anesthesia time, with fractional time units usually pro-rated for shorter periods.

Per Centers for Medicare & Medicaid Services and American Society of Anesthesiologists guidelines, anesthesia time is defined as “…the period during which an anesthesia practitioner is present with the patient. It starts when the anesthesia practitioner begins to prepare the patient for anesthesia services in the operating room or an equivalent area...” [1,2]. This period typically includes the time during which the anesthesia team has taken over principal responsibility for the patient, often commencing when the anesthesia provider picks up the patient in the preoperative area and transports the patient to the operating room. Notably, anesthesia time does not include the preoperative evaluation and examination, obtaining consent for anesthesia, nor any preoperative procedures such as nerve blocks or invasive monitoring catheter placements, some of which may be billed separately.

Modern computerized anesthesia information management systems allow for recording the beginning and end of anesthesia time within the electronic health record; however, these systems rely on the accuracy of clinician input [3,4]. While some investigations have failed to demonstrate evidence for systematic upcoding of anesthesia billing times [5], another recent study suggested that a significant proportion of cases, especially at academic centers, may have inaccurate anesthesia start times (ASTs) recorded, leading to loss of time units equivalent to hundreds of thousands of dollars in revenue each year [6]. Meanwhile, a 2011 study demonstrated that an intervention notifying clinicians of aberrantly documented ASTs with electronic charting alerts can improve the rate of adherence and therefore increase reimbursement [7].

In the present manuscript, we describe the conduct and results of a departmental quality improvement initiative in which we implemented a monthly communication informing anesthesia providers about AST documentation guidelines. As the outcome of the initiative, we observed trends in AST accuracy and estimated economic losses within the department over a one-year period.

This article was previously presented as a poster at the Yale New Haven Hospital Safety, Quality, and Experience Conference on June 2, 2022.

## Materials and methods

This was an internal quality improvement project performed in the Department of Anesthesiology at Yale New Haven Hospital (YNHH), a large, academic tertiary care center. The Yale Institutional Review Board (IRB), which was consulted prior to the study's start, recommended that it qualified as a quality improvement project and was exempt from further IRB oversight.

Anesthesia providers at YNHH, including residents, attendings, and Certified Registered Nurse Anesthetists (CRNAs), are mutually responsible for documenting the AST followed by the patient in-room time on EPIC (along with incision time, case closing, and other similar case milestones). As with documentation of medication administration, providers are able to subsequently adjust the recorded times to ensure accuracy. For the present quality improvement initiative, de-identified data were queried monthly from an internal electronic health record repository for all surgical cases at YNHH. Variables extracted included the difference in minutes between AST and anesthesia in-room time (“start to in-room time,” as charted in EPIC rounded to the nearest minute). Similar to previous studies, cases with a negative difference or more than 30 minutes between “anesthesia-start” to “in-room” were excluded from analysis [6,7]. For the present quality improvement effort, cases with 0-1 minute were presumed to be “likely underbilled” cases that were suspected of being charted incorrectly. In a separate empirical investigation of actual transport times, we documented that transports from preoperative areas to operating rooms at our institution required a median of 4-5 minutes. However, differences of up to 30 minutes were included in the analysis as certain cases at YNHH may require longer travel times, such as when the anesthesia provider transports a patient from an intensive care unit to the operating room.

**Figure 1:**
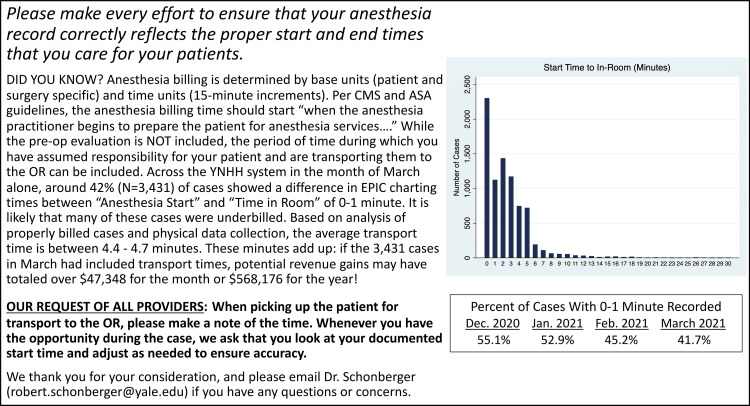
Example of a monthly educational email sent to anesthesia providers at the study institution CMS: Centers for Medicare & Medicaid Services; ASA: American Society of Anesthesiologists; YNHH: Yale New Haven Hospital; OR: Operating room

The project was run for a 12-month period beginning in January 2021. Data were first collected using all December 2020 surgeries for a baseline estimate of the percentage of cases that were likely to have been incorrectly charted, i.e., with a start to in-room time of 0-1 minute. Beginning in January 2021, an informational email was sent to all attendings at YNHH on the 15th of each month with an explanation of the charting rules for anesthesia and an update on the percentage of cases that were likely underbilled (please see Figure 1 for example email). Starting in April 2021, CRNAs were added as recipients, followed by residents in August 2021. At the end of each calendar month, data were queried for all cases and the subsequent email was updated with a graph showing changes over time.

A basic cost analysis of monthly and yearly revenue lost for “likely underbilled” cases was also included in these emails using the expected start to in-room time calculated from the average of properly charted cases. A weighted average of per-unit anesthesia reimbursement from private and public insurance, specific to case volumes and the patient population at YNHH, was utilized.

## Results

Baseline data

In December 2020, data for 6,967 surgical cases requiring anesthesia performed at YNHH were extracted (Table 1). Cases with a start to the in-room time of >30 minutes or <0 minutes were removed from analysis (N=90). Of the 6,877 remaining cases, 55.1% (N=3,790) had a start to the in-room time of 0-1 minute, which was considered “likely underbilled,” while 44.9% (N=3,087) had a start to the in-room time of >1 minute. Of the cases with times >1 minute, the mean start to in-room time was 4.42 minutes, with a standard deviation of 3.38 minutes.

**Table 1:** Comparison of monthly anesthesia start time data at the study institution * Cases with a recorded start to in-room time of <0 minutes or >30 minutes were not included in the analysis. ** Cases with a recorded start to the in-room time of 0-1 minute were considered “likely underbilled.”

Results by month (unit)	December 2020	June 2021	November 2021
Total cases (N)	6,967	8,401	8,199
Included cases* (N)	6,877	8,319	8,100
“Likely underbilled" cases** (N)	3,790	2,881	2,369
Proportion of cases “likely underbilled" (%)	55.1	34.6	29.2
Monthly cost estimate ($)	52,302	37,000	29,300

Baseline cost analysis

The total economic loss for likely underbilled cases was estimated by calculating the expected average number of time units lost per underbilled case using the mean start to in-room time of properly billed cases for December 2020 (4.42 minutes) with 15 minutes per time unit (rounded to nearest 0.1 unit), or 0.3 units per underbilled case. Based on 3,790 underbilled cases, the total estimate for money lost in December 2020 was $52,302.

Post-intervention trend

**Figure 2:**
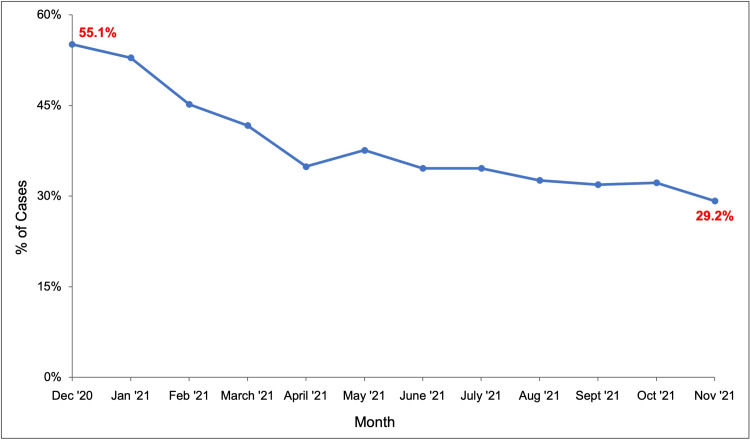
Percentage of monthly anesthesia cases that were “likely underbilled,” i.e., a start to the in-room time of 0-1 minute, over a one-year period at the study institution

After starting the departmental monthly email education initiative in January 2021, the percentage of underbilled cases was tracked for each calendar month (Figure 2) along with economic loss estimates. The proportion of underbilled cases showed a downward trend over the course of the project, decreasing from 55.1% in December 2020, to 34.6% in June 2021, to 29.2% in November 2021. The economic cost estimate for underbilled cases decreased from $52,302 in December 2020, to $37,000 in June 2021, to $29,300 in November 2021 (Table 1).

## Discussion

In this departmental quality improvement project, we utilized a simple monthly educational email sent to all anesthesia providers within the department to improve documentation of ASTs. We observed a clear downward trend in the proportion of cases that were “likely underbilled,” i.e. had an “anesthesia start” to “in-room” time of 0-1 minute (and thus unlikely to include patient transport times from the preoperative areas), during the course of the intervention. Furthermore, there was an observed decrease in the monthly economic loss estimates.

Some prior studies aiming to improve start-time documentation have utilized more involved and personalized techniques, such as automated alerts in the electronic chart during cases [7]. To our knowledge, this is the first study to show that a simple, department-wide educational monthly email on the topic can improve start-time documentation. Unlike real-time personal alerts, our described intervention achieved excellent results without the risk of interfering with providers during their active care of patients. The described approach also has several other advantages, including ease of distribution, minimal infrastructure requirements, and lower cost. Other work using email feedback for quality improvement is ongoing and typically relies on a robust clinical informatics infrastructure [8]. Groups such as the Multicenter Perioperative Outcomes Group [9-13] quality improvement and research consortium have a widely deployed monthly email quality improvement program that has demonstrated incremental savings within the Michigan Surgical Quality Collaborative [14] as well as improvements in the process of care metrics such as train-of-four monitoring [15]. Considering that properly recording the first few minutes of anesthesia time spread over thousands of cases a month can lead to large reimbursement gains, we believe departments across the country could benefit from this simple intervention, especially at a time when reimbursement rates face ongoing downward pressures and as inflation continues to erode the value of even stable reimbursement levels.

We do wish to point out several limitations to this quality improvement initiative. Since it was purely observational, it is impossible to conclude the causality of our intervention. It is possible that other factors unknown to the investigators were simultaneously at play and influencing provider charting behaviors. Furthermore, our analysis provided overall departmental-level performance with a focus on overall trends. Monthly differences in case mix or total number of cases at different sites might have skewed the results, but we would expect such variation not to yield the steady improvements that were observed. Additionally, while our intervention was effective at a single center, further work would be needed to assess generalizability to other healthcare systems [16].

Similar to prior studies, we included cases with up to 30 minutes between anesthesia start and in-room time in our analysis [6,7]. While we found that most transport times from preoperative areas to the operating room at our institution take 4-5 minutes on average, there are several situations in which anesthesia providers take principal responsibility and travel with patients from more remote locations, such as the intensive care unit or the emergency department. We thus utilized a 30-minute cutoff while recognizing that a small number of included cases may actually be examples of overbilling.

Finally, a portion of the “likely underbilled” cases was in fact properly documented. Several situations indeed do lead to anesthesia start times immediately prior to or simultaneous with room-in times, which ideally would be documented by providers in the medical record (using a comment or quick note feature). Examples include emergency cases when the first anesthesiologist contact with the patient might occur as the patient is being transferred into the operating room. Other cases that may in fact be correctly billed could include high-volume procedural areas where preoperative waiting rooms are directly adjacent to their operating rooms. Still, our empirical analysis of actual transport times demonstrated that overall average transport times are typically greater than 0-1 minute. Thus, while we do not comment on any individual case as an example of definite underbilling, we remain confident that in the aggregate, the majority of cases with 0-1 minute transport times are consistent with incorrect charting. Moreover, the mere fact that our observed proportion of “likely underbilled” cases steadily dropped during the intervention lends further credence to the assertion that providers realized their charting inaccuracies and worked to correct them.

## Conclusions

Anesthesia billing in the United States incorporates time-based units for reimbursement calculations. Thus, accurate documentation of the start and end time of anesthetic care by providers is critical to ensure proper billing compliance and maximize potential revenue. The present report demonstrates that a simple department-wide monthly email educational intervention correlated with an improvement in anesthesia start-time documentation. Furthermore, there was a reduction in estimated economic losses due to underbilling over the course of the study period. These findings could be of interest to other institutions seeking to improve their documentation accuracy with a low-cost and collective approach.
